# Using Community-Partnered Participatory Research to Value the “Community Lens” and Promote Equity in Community–Academic Partnerships

**DOI:** 10.1089/heq.2023.0096

**Published:** 2023-09-13

**Authors:** Hafifa Siddiq, Felica Jones, Zoe Magnes, Juanita Booker-Vaughns, Angela Young-Brinn, Clarence Williams, Madeline Washington, Etsemaye Agonafer, Olga Solomon, Adrian Oliva, Kenneth Wells, MarySue V. Heilemann

**Affiliations:** ^1^ College of Nursing, Charles R Drew University of Medicine and Science, Los Angeles, California, USA.; ^2^ UCLA David Geffen School of Medicine, Division of General Internal Medicine and Health Services Research, Los Angeles, California, USA.; ^3^ Healthy African American Families-II, Los Angeles, California, USA.; ^4^ Community Faculty, Charles R. Drew University of Medicine and Science, Los Angeles, California, USA.; ^5^ Department of Health System Science, Kaiser Permanente Bernard J. Tyson School of Medicine, Pasadena, California, USA.; ^6^ USC UCEDD at Children's Hospital Los Angeles, Los Angeles, California, USA.; ^7^ Keck School of Medicine of University of Southern California, Los Angeles, California, USA.; ^8^ UCLA Jane and Terry Semel Institute for Neuroscience and Human Behavior, Los Angeles, California, USA.; ^9^ UCLA David Geffen School of Medicine, Fielding School of Public Health, Los Angeles, California, USA.; ^10^ Greater Los Angeles VA Health System, Los Angeles, California, USA.; ^11^ UCLA National Clinician Scholars Program, David Geffen School of Medicine, Division of General Internal Medicine and Health Services Research, Los Angeles, California, USA.; ^12^ UCLA School of Nursing, Los Angeles, California, USA.

**Keywords:** community engagement, community–academic partnerships, community-partnered participatory research, community-based participatory research, health equity, equity

## Abstract

**Background:**

Community input is crucial for identifying characteristics necessary for equitable, sustainable community–academic partnerships (CAPs). A November 2021 conference, honoring the late Dr. Loretta Jones and the Community-Partnered Participatory Research (CPPR) model, was held to gather input for designing a learning institute for community members as co-equal partners with academics in research, program, and policy initiatives. This created an opportunity to explore attendees' perspectives on challenges and opportunities related to CAPs with special focus on promoting equity.

**Methods:**

Institutional Review Board approval was obtained. Five break-out discussion group sessions were conducted in November 2021 co-facilitated by both an academic and a community leader. After consent, discussions were recorded and transcribed. An iterative procedure for collaborative-group-thematic-analysis was developed. The six-phase process included rigorous coding, discussion, comparison of data with data, and development and refinement of themes and subthemes.

**Results:**

A total of 38 racial-ethnically diverse participants volunteered from the total conference audience of 62 community and academic partners from various sectors including community-based organizations, health care, social services, academia, or policy within Los Angeles County. Analysis led to development of three themes: Being cautious with the extractive tendency of academia and the need for anti-racism within CAPs; Leveraging community power to resist the top-down lens of academia; and bridging two worlds through an equitably structured table.

**Discussion:**

Participants described optimism about the future uses of CPPR to enhance CAPs, and the need to address barriers to equitable partnerships owing to unequal social contexts and entrenched power dynamics. Implications include addressing racism, evaluating financial equity in partnerships to promote accountability, and mentoring community leaders to promote equity.

**Conclusion:**

Use of a “community lens” for developing sustainable, equitable CAPs is crucial to promote accountability and to responsibly implement authentic CPPR.

## Background

Health inequities continue to perpetuate in the United States as a result of structural racism. Community and academic partners have increasingly advocated for community-based participatory research (CBPR), which emphasizes collaboration and equitable involvement of community members and stakeholders in the research process to address health inequities.^1–3^ A notable variant of CBPR is the Community-Partnered Participatory Research (CPPR) model, which facilitates the development of equitable partnerships through a three-phase process (Vision, Valley, Victory), and provides a robust framework for community and academic collaborations aimed at addressing health disparities.^4–6^ The success of this approach hinges on partners who are not only well-versed in the model, but also representative of their communities and committed to utilizing scientific research in their collaborative efforts.^7,8^

Central to addressing health inequities is the establishment of community-academic partnerships (CAPs), which often involves predominantly White academic institutions working in tandem with communities of color. The fostering of CAPs is essential to responding to calls for anti-racist approaches within public health,^9^ and to policy development that will lead to the advancement of health equity. Scholars have called for a more robust conceptualization of CAPs and development of evaluation tools to measure success of such partnerships,^10^ because a clear consensus on the defining characteristics of equitable and sustainable CAPs remains elusive. However, it is imperative that the development of any CAP conceptualization or CAP evaluation tools be informed by community perspectives.

An opportunity to promote equity in CAPs in the context of CPPR occurred because of a unique partnered conference series in November 2021, the virtual Community Leadership Institute for Equity (C-LIFE) conference. The conference aimed to honor the pioneering work of late Dr. Loretta Jones in the development of the CPPR model, to build upon the work of Communities for Wellness Equity^11^ and to combine frameworks of CPPR and anti-racist approaches for the purpose of enhancing CAPs. Thus, attendees across multiple sectors from Los Angeles County were invited to share challenges, barriers, and opportunities for equitable CAPs. We conducted break-out discussion groups during the conference to gather input for designing C-LIFE, a learning institute for community members as co-equal partners with academics in research, program, and policy initiatives. The purpose of this study was to explore and analyze the perspectives on challenges and opportunities related to CAPs with special focus on promoting equity.

## Methods

After receiving approval from the University of California, Los Angeles (UCLA) Institutional Review Board, a CPPR approach was used to foster partnership in the research process. This included collaboration in the development of discussion questions, facilitation of discussion groups, data analysis, and dissemination of findings. Our study team included both academic and community partners, with the majority having extensive experience with CPPR as part of the Los Angeles community. Many had a history of working together with an organization serving African Americans; this included a postdoc fellow with a deep commitment to CBPR.

The study was informed by the Public Health Critical Race Praxis (PHCRP) framework that guides investigators to remain attentive to equity while carrying out research, scholarship, and practice.^9^ It led to a race-conscious iterative examination of community leaders' perceptions of barriers and facilitators to successful, effective CAPs. Knowledge production with the PHCRP challenges the historical racial biases embedded within public health, medicine, and other health fields, which have often overlooked the intellectual contributions of people of color and the consideration of racism as a crucial determinant of health.^9^

### Sample and data collection

At the end of the conference in November 2021, attendees who were over the age of 18 and who identified as community members were invited to participate in voluntary break-out discussion groups as part of this research study; most identified as being from Los Angeles. Study information was shared, informed consent was obtained, and then participants joined break-out rooms where discussions were co-facilitated by both an academic and a community leader. Discussions were recorded and transcribed for analysis (Table 1).

**Table 1.** Discussion Group Question Prompts

(1) What does community partnership mean to you?
(2) What does community partnership mean to your community?
(3) Think about an academic–community partnership you know of. How was it developed?
(4) When you think about that partnership, tell me what is and isn't working about it?
(5) How can we do more of what works?

### Data analysis

An iterative methodological procedure for a collaborative-group-thematic-analysis was developed.^12^ An inductive, latent-level thematic analysis was conducted to examine and interpret the data. The first phase involved reading transcripts multiple times and the second phase involved generating initial codes, using a variety of coding techniques. Process coding used gerunds to identify action in the data. Emotions and values were identified in codes and *in vivo* codes captured poignant expressions of meaning, preserving the words of participants.^13,14^ In the third phase, we examined recurrent codes and created “bucket themes” that were considered important by the research team.^14^ The fourth phase involved rigorously scrutinizing themes by reviewing and comparing them. The fifth phase involved clarifying each theme as distinct from each other, defining it and renaming it; the sixth phase involved refining themes to enhance coherence of meaning.^12^

## Results

Five break-out discussion group sessions were conducted in November 2021 with a total of 38 racial-ethnically diverse participants who volunteered from the total conference audience using prompts (Table 2). They represented various sectors including community-based organizations, health care, social services, academia, or policy within Los Angeles County. Analysis of data led to the development of three themes related to promoting equity (Table 3).

**Table 2.** Conference Participants' Characteristics (*n*=62)

Roles	
Community member	20
Faith-based organization	2
Community-based organization	19
Researcher	33
Educator	11
Policy	2
Stakeholder	8
Service provider	7
Community leader	12
Other	14
Highest level of education	
High school	1
Some college	2
Associates/trade/vocational	2
Bachelor's	17
Master's	16
Doctorate	23
Other	1
Race/ethnicity	
American Indian/Alaska Native	1
Asian	6
Black or African American	22
Hispanic or Latino	11
Native Hawaiian or Other Pacific Islander	2
Non-Hispanic White	13
Two or more races	4
Refuse	1
Involvement in research	
I have been a research participant in studies (completed surveys, participated in interviews or focus groups)	12
I have experience as a researcher	34
I have experience as a stakeholder (used personal experiences to provide researchers with guidance, advice, or consultation on their research)	22
I do not have research experience	9

**Table 3.** Themes, Subthemes, and Exemplar Quotes

Theme	Subthemes	Exemplar quotes
(1) Being cautious with the extractive nature of academia and the need for anti-racism within CAPs	Exchange of resources	“I think community partnership, what it means to me in the work that I do is really about a resource exchange and that communities really honoring and acknowledging and valuing the resources that they hold in ways that equitably distribute resources within a partnership.” “When I hear and use the term ‘partner,’ that there is a long-termness to it. It's not transactional. And I feel like so much of the work that myself and other researchers that were pressured to do, is very transactional. It's exchanges. Where the partner is someone who has your back, you have their back, it's a relationship where there's no need to use your voice necessarily. It's really your presence.” “Partnership is an evolving relationship whereas power is shifting because there is nobody who doesn't have power. A scientist has power, a community has power. People in the community have power on each other.” “I differ, I don't think it's relative. I don't think it has a timeframe on it. I can partner with you for transactional, or I can partner with you for ongoing. It's a collaboration, a sharing of bringing thoughts, bringing work, bringing, working towards a conclusion of ideals of a solution for an idea. And that can change it. It doesn't… My partnership with institution might be this, my partnership with a community might be that. But partnership is a broad term, but I think it always will deal with collaboration, working together. There is also the side of the academics exploiting communities by means of graduates. The students would like to do a three-month research [project] and we have the nonprofit and interest to get some work done. And so, it's a mutual exploitation, if you want. But it's not real work… All sides are benefiting, but nothing is happening after that.”
	Context of racism	“One of the pieces of this systemic racism has to deal with this, this whole idea of sharing power… [academics] are scared to share the power, which means that certain doors kept close for us to have the ample kind of representation that we need to be more actively engaged in help us to solve a lot of problems.” “The anti-racism part, what is that? How do we identify that? Because you cannot force people to change, but it's embedded throughout the government, the legal system, and how we move and have our beings move throughout the country. [Racism is] something that must be identified by scholars and thinkers and community alike because we would like sustainable lives, because we saw one of the definitions of racism, that racism actually causes early death. That it's causing early death in particular communities, and especially the Black community.”
(2) Leveraging community power to resist the top-down lens of academia.	The “community lens” and sources of power through lived experiences	“My specialty has been in housing and employment, but many of the tables that I go to and talk about how to deal with prevailing issues and, community, and how African-Americans are so disproportionately represented in so many categories that the problem that I see is that everybody views [a] situation, ideal with life, through a certain lens.”
	The “academic lens” and navigation of unwelcoming spaces	“And then our value is still questioned after we get to that table, even though we're coming to that table sometimes equally yoked in terms of academia. But other times we bring with us our degrees a life experience within those communities that they have never lived. And so therefore we bring more to the table.” “I'm going to share from my perspective as a faculty member and researcher… The main thing I think about is how I'm in this place where I'm constantly trying to navigate where I don't really belong. So, from the outside I look like a professor, but within the academic setting you're looking at me like, who are you? ‘We're not trying to address racism, and why are you here? You're not really one of us. What you do, that's fine. That's on the margins.’” “I carved out my own place of what this work would look like. It did lead me to get a master's degree. My colleagues in institution at the time… they said, ‘Oh, well, you might be interested in doing some study in early child development.’ So, I did a two-year certificate in early child development and administration. And what that did, it opened-up my language, because our [community] work mission is specifically on maternal and infant health, black maternal and infant health. So, it opened-up my language around human development. It opened-up my language around developmental scholars. It opened-up my language to really have a broader view.” “What I have discovered, and I don't care if it's a high school student, an undergrad, a master's student, doctoral student, post-bac, or post-doc, that one of the challenges, and I'll say specifically for those of us who come from the black community and want our way up, where we're intrigued by this work and want to do the work. But we lack the experience of saying, ‘I'm enough.’” “Now I wear a doctor hat, I wear a researcher hat, I wear a medical educator hat, but being the daughter of an immigrant father, me, myself, being English as a second language, a mother who was first generation in college, both of whom came from poverty. For me, [I] experienced imposter syndrome. And it's a struggle for me even today when I come into a room… we need to build confidence in leaders.”
(3) Bridging two worlds through an equitably structured table	Intentionality and humility, open to different perspectives	“Community-based partnerships establish trust between two groups or more multiple groups that, you know, historically haven't maybe had a trusting relationship, or haven't always seen eye to eye. Their goals have not always been aligned. But this kind of model is a way that we can move forward and work together and to build that trust, seek solutions… because [even if] someone is an expert doesn't necessarily make what they have to say or the advice that they have to give palatable to the people that they're trying to [reach]… it's a way for people to come together as equals and build that trust where maybe trust wasn't there before.” “I don't think that it takes a special person, it takes people that can connect together on a human level to do that collective good, to get enough people to participate and to understand them and where they're coming from.”
	Before establishing the partnership, build trust through time, humility and transparency	“We need to be able to see the level of transparency to give us an idea if we're able to trust you. And that only happens through established relationships… put the cards on the table, reveal yourself, state your interests, give us an opportunity to let us know that you're genuine, and that you're going to want to work with us, but also learn from us, and would like us to contribute to the work.” “Community-based partnerships establish trust between two groups or more multiple groups that, you know, historically haven't maybe had a trusting relationship, or haven't always seen eye to eye. Their goals have not always been aligned. But this kind of model is a way that we can move forward and work together and to build that trust, seek solutions… because [even if] someone is an expert doesn't necessarily make what they have to say or the advice that they have to give palatable to the people that they're trying to [reach]… it's a way for people to come together as equals and build that trust where maybe trust wasn't there before.” “If you really want to be effective in working with any population, whatever your lens is you have to turn it off and look at things with a fresh set of eyes, to try to see, see a view point from someone else's view and not put your own spin on something.” “I don't think that it takes a special person, it takes people that can connect together on a human level to do that collective good, to get enough people to participate and to understand them and where they're coming from.” “I remember being on grants that the principal investigator would be African American, and that for the most part was considered that it was community participatory research, and that even though the community members weren't really involved with developing research questions or the methodology… I think the only way to be effective is if we include community and the development for all stages of research.” “Be patient, to create those relationships with us. Why not when you get those first thoughts of creating that first research project or that first grant, invite us to the table to talk about what the content of that should be, what the questions of evidence should be, let us be in on the embryonic process.” “…working behind the scenes with institution and institution have brought a lot of awareness to me, and I know that helps the community as well. And if [academics] keep sharing [their] testimony that information is going to link in with the community, they are going to bring more to the table.” “I think it's essential that those involved with research already have relationships established with the organizations, you know, that they've worked together and that they know the communities before they launch a research project.” “Trust comes from transparency and honesty, and so that's what is needed.”
	Critically assess who is and isn't in the room, and raise awareness to engage/bring in more community perspectives	“Because now we're looking for those voices within that are going to be the change agents to bring us to the table and to say who's missing from this thing as you're moving up the ladder.” “The look of these rooms has got to change. And the black people that are sitting in the room, can't keep being exported by us. He made that reference in that room. We were only in that room because he and Loretta escorted us. And so that part, decision-making, because we are not the decision makers, so when you are not a part of decision making, you're not a part of the play. Then you're here not relevant at the men or women in the box. Because you're not there at the beginning when you're able to help make the decision.” “I have come to some understanding by working behind the scenes with institution, you know, my sister and having conversations with the family, different things. But, you know, you find that a lot of times things are in the community that the community doesn't know about. They have never been invited to the table or asked if it's okay to bring it to the community. And it just, just in the unknown.” “I didn't know that these community departments even were doing things for the community in the background, until, I started working with my sister and my brother, you know. I believe personally that there's a lot of things that the community is just in the unknown about.” “And they don't know how they—they believe that, I think that they believe, that they don't have any part of the decision-making for their community. They think it's something that is thrust upon them, you know.” “Break that barrier and when you go into the community and let them know that what they have to say matters. I think that's where trust comes in at. When they learned that they are a part of the decision-making because, you know, at this point right now, you know, I think a mass majority believe that they just don't have any input.”
	Ensure a welcoming, supportive environment	“Not just sort of getting people to the table, but when they're at the table, feeling like they deserve to be, knowing that they deserve to be there. Speaking their truth so that if they don't get it in the moment, they have somebody, a voice, a powerful voice. And that was what Ms. Loretta was for so many of us. She was that voice that says, ‘I love you unconditionally. I believe in you unconditionally. You deserve to be here. I'm bringing you here, and I'm going to make sure you stay here, and that I've got your back.”
	When establishing the partnership, align mutually beneficial goals, map out assets, and be transparent about the agenda and expectations	“It was in the development of any sort of proposal, that by including and building up from the inception of those earlier projects, it allows for a rapport and a dynamic and a trust to be able to support each other's work at no matter what stage of a project the partner might be in. And so, I think that that is really important.” “In thinking about the relationship between the researcher and the community-based organization, is that it's mutually beneficial. Because when I as a partner help you as a researcher, you help me as a community partner. And it's this bi-directional, that seeing you succeed is going to help me succeed and that helping your researcher with promotion and tenure is going to allow for that dynamic of getting those CPPR grants and getting those projects off the ground and just supporting each other in that way, and seeing when you succeed, I succeed, kind of thing too.” “Ask questions… So, what are the needs or what are the strengths that need to be developed? When you think about partnerships that you sat at the table with, how was your voice leveled in those spaces? Was it balanced? Was it equitable? How do we create a training institution that talks about these kinds of balances and checks and balances that we train up? Not just the community, but the academic and everyone else on the other side, so they can understand that this is the highway that we're working on.” “We need to see the level of transparency that you have took to this and to give us an idea even we're able to trust you. Before you even start talking about you, don't just need to come [and] see what's happened in my neck of the woods. Put the cards on table, reveal yourself and state your interest and give us an opportunity to let us know that you're genuine. And that you're going to want to work with us with not only just work with us, but you also want to also learn from us. And will like for us to contribute to the body of work that's about to happen.”
	Promote transparency around the budget and fair compensation	“I'm just thinking if it's an academic institution that it needs to have partnered with community-based organizations and brought them in financially, in terms of funding, that's important. There has to be some financial—not incentive—but there has to be a financial gain as well for the community-based organizations, which are basically struggling financially.” “There's always a need for the community organizations to have funds, to be able to operate, you know, to have enough for, you know, just to operate day to day.” “One of the things at agency that we tell our partners or people looking to build partnerships, either with institutions or with community-based organizations, is what does the budget look like? That's the biggest resource when you're thinking about coming into a partnership where there is a power dynamic of financial distribution of resources of whether that's land or facilities or access to different networks, and even just relationships with funders and things like that.” “I think that's one thing that I see that even in dynamics where you're trying to have a non-hierarchical partnership and you're trying to build partnerships with community members from an institution point. If you're still the one that is accountable to the funder and accountable to managing the finance that, and you haven't addressed that power dynamic, then there's like an inherently paternalistic kind of dynamic there. And so, I think that it takes a lot of intentionality.” “What does the budget look like? That's the biggest resource when you're thinking about coming into a partnership where there is a power dynamic of financial distribution of resources.” “… [funding for communities] should be implemented and be a part of the design and every strategy, and not necessarily an afterthought.” “I'm just thinking if it's an academic institution that it needs to have partnered with community-based organizations and brought them in financially, in terms of funding, that's important. There has to be some financial, not incentive, but there has to be a financial gain as well for the community-based organizations, which are basically struggling financially.”
	Identify barriers to how community input will not be used	“Looking into those systems and understanding the process that's causing the barriers and through for you to be the voice of change within the system, because you can come out as that individual doc and say, I want to do this research go in this particular, but there's always going to be those barriers for you on your end.”
	Dissemination to the community and training community to disseminate to others	“You need to see results, some form of result. If it's for the group that I bring to the table, I want to see progress being made. If it's for the group that you're bringing to the table, I'd like to see the progress that you are making for yours. I had to remove myself due to poor health, so I didn't get to see the completion of it. But from the little bit that I do still hold, touch with, I think that everybody is doing just that. They're finding their own success coming out of that partnership.” “I think it's important to acknowledge and celebrate the little things that are accomplishments. Because so much of what we accomplish, we won't see it. It's not a widget on assembly line. We can't count it under the year. So, we have to say, ‘Oh, we made a decision and wow, this is something to celebrate. [Celebrate] not just the completion. It is success’. You need to have a win in order to boost your stamina to continue. You don't want to be the little mouse in the wheel that goes round, and round, and round, and round, and round, and round, and round. You want to see some form of success. That does not mean completion.”
	Assuring there are policy implications	“There has to be hopes of changing policy.” “This [project was] the first time that community had been apart at this level of the process of shared decision. So yeah… everybody dived in to help me to make this a model that could be utilized. And so, we did. Even me being a part of writing the curriculum for shared decision making in emergency medicine for undergrad and graduate ended up a policy.”
	Sustaining the project and the relationship	“Without sustainability implemented in the design, that's really going to hinder, you know, not just community participation, but also community trust.” “Partnership has been just such an example of where… It truly is a relationship where, it's not just one project, they've been on multiple projects together over the years through different jobs and research stations and things like that, to where it's just like, hey this is what I'm working on, or this is a grant or funding opportunity. Where do you want to fit in? And just always deferring to each other and always lifting each other up. Thinking about like a partnership in a relationship it's on that level.” “How do we build the community's bank instead of individual large projects, little projects, different projects? We are a whole community. We are all in need of something. But if we had a larger bank to pull from, I think we would all get something. This separation, is it a learned behavior or a taught behavior or an ongoing behavior? We have this strong need to separate this cause out from that cause. It's all a community cause.”
	Consider long-term relationships.	“I think that with the partnership between agency and institution, what works about it is that they're always uplifting each other's work, even if it's not something that they might be directly involved in. They're always connecting each other to these different projects and these different funding opportunities. And really, I think it's a co-learning and a co-mentorship and a co-production of the research questions, the dissemination plan, the implementation plan, and really deferring to each other with humility. I think that's something that I've witnessed.” “And just being there to uplift each other even if it's a project that we're trying to apply for, providing letters of support for community partners, providing in kind staff support for your community partner, providing connections with your academic partners for other community leaders and organizations or opportunities to expand their community engagement. But I think the main thing is that it's not just the continual connection that has built trust and trustworthiness over decades of time.”

CAPs, community–academic partnerships; CPPR, Community Partnered Participatory Research.

### Theme 1: being cautious with the extractive tendency of academia and the need for anti-racism within CAPs

Participants emphasized the importance of recognizing power dynamics between the community and academia and the need for an anti-racist approach. This led to two subthemes.

#### Subtheme: exchange of resources

They expressed concerns regarding the extractive tendencies of academia and the need for equitable, resource-sharing partnerships like CPPR approaches. A two-way sharing of resources was deemed essential in preventing an “extractive, transactional kind of partnership,” which was perceived as inequitable. A participant raised a strong caution saying, “it's a relationship for me. Don't mine gold out of my community, for someone else to profit.”

Participants noted that CPPR fosters open discussion so people can share what they really think. However, without commitment to the proper implementation of community-partnered approaches, academics often prioritized data over the needs of the community that hindered the development of genuine and equitable partnerships. They emphasized that equitable partnerships between community and academia should not be exploitative, but rather foster an equal exchange of resources. Community resources were variously described as encompassing experiential knowledge, people power, time, connections, and equipment needed to carry out the project. Other key resources included funding, compensation for staff effort, and infrastructure.

#### Subtheme 2: context of racism

Participants voiced a tension around acknowledging racism in academia and the need to address “deeper social problems” to solve health issues. Some reflected on the past and how this was not historically addressed. Others pointed to recent Black Lives Matter protests that sparked a renewed awareness of racism even within academia, which “caused people to really think again how we do anti-racism in our group and so I'm optimistic.” Another noted that “people are waking up and hearing our voices, and they understand the importance of inclusion.”

### Theme 2: leveraging community power to resist the top-down lens of academia

The inherent strengths of communities are addressed in two subthemes. The first highlights the community point of view, a community lens, for focusing on CAPs and the second addresses the lens through which academia perceives CAPs. The latter includes the need for community members to navigate unwelcoming spaces.

#### Subtheme: the “community lens” and sources of power through lived experiences

Participants rejected the notion that they needed to be empowered, saying, “We have power, we have a great deal of power.” They identified how they use a “community lens” that is informed by “being in the trenches” and possessing a “deep understanding of the needs of the community.” A participant emphasized the power of lived experience and informed intentions as, “witnessing issues faced with and wanting to do something about it.” Another participant highlighted the powerful history of community efforts despite limited resources as “making the most of what was available for us (at the time).” There was a call for health care systems to support “efforts that have already been developed within the community” because, “if you really want to fix a problem… consult us!”

When determining a solution, participants insisted that CAPs should “value us enough to trust us enough, to really, to be able to solve a problem.” Although mentorship from key community leaders, such as Dr. Loretta Jones was highly valued, more mentorship in research engagement was needed as community members reported a tremendous desire to cultivate a new generation of community leaders who could have an impact on research.

#### Subtheme: the academic lens and navigation of unwelcoming spaces

The top-down lens of academia was perceived as perpetuating power imbalances within CAPs. Academic credentials were often valued over lived experiences and participants critiqued this as a form of gatekeeping; they felt such qualifications should not be a prerequisite for involvement in CAPs. They acknowledged the benefits of academic credentials and described education as “a doorway that opens up opportunities to carve out a specific expertise.” However, community members with academic credentials, particularly Black academics, were “still questioned at the table” although they brought value that academics without community experience could not bring.

Others felt tokenized when academics assumed that one community member represented the entire community instead of including a broader range of perspectives. Even at the table, gatekeeping or omission of ideas from the community led to marginalization of their input; ultimately, these experiences led to avoidance or mistrust of academic researchers.

A participant described the frustration of having to “fight… to be relevant in a space that they say they can't do the work without the community.” These led to the perception that the “community” was “less expert.” Such disempowering experiences led participants to call for more equitable collaborations that truly benefit the communities they aim to serve.

### Theme 3: bridging two worlds through an equitably structured table

Twelve subthemes were identified (Table 3) reflecting hope and enthusiasm about the path forward using CPPR to build trust between the two worlds, community and academia, worlds that “haven't always seen eye to eye.” To do the work, “the table” would need to be recognized as a place of power where decisions are made, often representing White academic institutions. However, to achieve equity, participants recommended addressing power imbalances by critiquing the initial set-up of the table and identifying who is and isn't present in decision-making spaces. Participants recommended promoting an inclusive and welcoming environment in these spaces, nurturing a partnership over time, and ensuring transparency and continuity.

Participants also emphasized that developing an equitably structured table will require openly acknowledging the role of financial power dynamics, including how funds are allocated, and transparency about the budget. Successful CAPs and projects were seen as requiring investments of time and financial resources over time, building trust between groups with historically divergent perspectives, and respecting community voices in decision-making spaces. Structural issues would need to be addressed to promote equity in CAPs which is why CPPR was considered to be crucial to fuller understanding at every level. A participant noted, “It's important that when we're talking about these academic and community partnerships, that we also realize how they operate within these larger systems.” By making these recommendations, participants hoped to promote equitably structured “tables…for people to come together as equals and build that trust where maybe trust wasn't there before” through meaningful, long-lasting CAPs (Table 4).

**Table 4.** Outline of Recommendations to Support Equitable Community Partnerships

Phase	Recommendations
Partnership development phase	Address power imbalances through humility and intentionality and be open to different perspectives.
	Before establishing the partnership, build trust through time, humility, and transparency.
	Critically assess who is and isn't in the room, and raise awareness to engage/bring in more community perspectives
Establishing the partnership	Promote transparency with budget and fair compensation
	Ensure a welcoming and supportive environment
	When establishing the partnership, align mutually beneficial goals, map out assets, and be transparent about the agenda and expectations
	Transparency through the establishment of an MOU, and around the budget and fair compensation
Maintaining the partnership	Incorporate ways partnerships can affect policy
	Identify the small wins
	Sustaining the relationship beyond the research project

MOU, Memo of Understanding.

## Discussion

### Summary of main findings

Although rarely examined, the perceptions and experiences of community partners with academic institutions have important implications for promoting successful CAPs, public health research, and designing C-LIFE, a training institute for community leaders as coequal partners. Our findings reveal optimism among community leaders about the future uses of CPPR to enhance CAPs, and the need to address barriers to equitable CAPs owing to unequal social contexts and entrenched power dynamics.

In alignment with the principles of CPPR, an effective and successful CAP is equitable for both sides. This requires deliberate, intentional reflexivity to recognize and rectify historically skewed power differentials. For communities to be considered true partners in research processes, academic teams must fully partner with communities and be accountable to them. When evaluating the effectiveness and equity of CAPs it is crucial to assess the level of value placed on community voices by academics and actions taken to address systemic racism within the context of CAPs.

### Implication 1: addressing racism in the context of CAPs

Structural and interpersonal racism in academia must be addressed to promote equitable CAPs.^15^ Power imbalances, including the extractive nature of academia, hinders the development of genuine and equitable partnerships. While CBPR approaches have been used for decades, the need to re-examine and critique collaborative processes is ongoing. Mistrust, miscommunication, and weak relationships are common in CAPs,^10,16^ and these issues are rooted in the social context of race and racism.^17^ Experts agree that more accurate and more complete understandings of racism are urgently needed, including in relation to science. Our findings support the urgent need for academia to be deliberate about addressing structural and interpersonal racism owing to its influence on the processes and outcomes of research partnerships.^18^

Although scholars have identified the need for more diversity initiatives,^19^ there is also a need to be cautious to avoid tokenism or omission of minoritized voices within academic settings. Racism can exacerbate problems rooted in power differentials, but these problems could be addressed through deliberate and actionable steps; such steps require critical reflection and assessment of CAPs including issues of time, humility, and financial investments.

### Implication 2: evaluating financial equity in partnerships to promote accountability

Although previous research has identified characteristics to assess CAPs, our study expands the literature to emphasize community capacity for leadership and attention to financial power dynamics.^1^ There is a need to be mindful of dynamics within CAPs, particularly related to financial resources; transparency in relation to funders to reduce imbalances. Although institutions, especially established White institutions, historically and currently benefit from federal research funding with high negotiated indirect cost rates that support their infrastructure, community-based organizations often struggle to provide services to communities and may bear the burden of unfair compensation for research involvement. Infrastructure must support financial equity for all partners. Future community-based collaborators need to be identified and equipped to lead through training that includes skills for equity and financial accountability.

Moreover, disparities in research funding and unequal distribution of funding and resources between academic institutions and community-based organizations may have implications on under-resourced communities. For example, a recent study focused on the distribution of research funding in Fiscal Year 2020 by organization type; results revealed that medical schools received higher levels of funding than other institutions, with the top 10% receiving 70% of research funds. Inequalities among organizations were much greater than inequalities among Primary Investigators (which showed the top 1% of funded PI's were more likely to be in later career stages, to be White and male, and to hold a Medical Doctor degree).^20^

Moreover, affluent institutions, including medical schools, that claimed to value supporting underserved communities often resided mere streets away from impoverished neighborhoods suffering from discrimination and segregation, yet the schools were cited as tokenizing the involvement of minority representatives. This serves as an example of the extractive nature of academic institutions that mirrors a colonialist mentality with communities being mined for research data by academic institutions that benefit from substantial funding while community-based organizations receive minimal or no benefits.

### Implication 3: mentoring community leaders

There is a need to promote mentorship and capacity-building within communities to strengthen their roles in CAPs. Community members desire involvement in research through mentorship. Bidirectional learning that results when academic researchers and community members conduct research together, shoulder-to-shoulder, is crucial for working toward health equity. Building teams of community leaders who are equipped to work in partnerships with academia should include a process of training and mentorship that: recognizes the (1) value of life experiences, (2) strengths of community leaders as advocates with unique lenses that are vital to making impactful change, and (3) need to tailor training and research involvement to enhance and expand existing abilities. Indeed, well-designed, collaborative community leadership training programs that address and mitigate the detrimental challenges likely to be faced by community leaders in academic settings hold promise for creating and sustaining innovative, productive, and effective CAPs.

## Strengths and Limitations

Our study had several strengths including our community-partnered approach to the entire study and data analysis process. In addition, the data analysis team co-facilitated discussion groups and community feedback was obtained at each stage of the research process including the development of the findings and identification of implications. However, our study has some limitations. Participants in our study were primarily individuals who had attended a specific conference focused on CPPR, and thus, their perspectives may not represent the full range of experiences and opinions of community leaders in different settings. Not all participants shared their level of experience or background in CAPs and those with more experience or who had negative experiences with academics in general may have been more vocal or willing to share their experiences during discussions.

## Conclusion

The study's findings highlight the ongoing barriers to equitable CAPs, despite community leaders' optimism about CPPR's future directions. Despite decades of advocacy for community partnerships in research, significant barriers to truly equitable CAPs persist and warrant ongoing scrutiny. These findings underscore the importance of examining and valuing community perspectives on CAPs to promote accountability and to responsibly implement authentic CPPR. Addressing systemic racism and power imbalances within CAPs is crucial for academia to advance health equity in collaboration with communities. To do so, employing frameworks such as the PHCR framework can help to confront these challenges and foster a more equitable environment for collaboration. By acknowledging and addressing these barriers, we can create more just and effective partnerships that drive meaningful progress toward health equity.

## Abbreviations Used

C-LIFE: Community Leadership Institute for Equity
CAPs: community–academic partnerships
CBPR: community-based participatory research
CPPR: Community-Partnered Participatory Research
CRECD: Clinician Research Education and Career Development
MOU: Memo of Understanding
PHCRP: Public Health Critical Race Praxis
UCLA: University of California, Los Angeles
